# Prevalence of Glutamic Acid Decarboxylase (GAD) Antibody Positivity in Young-Onset Diabetes: A Retrospective Study From Northeast India

**DOI:** 10.7759/cureus.111894

**Published:** 2026-07-01

**Authors:** Pranjal K Dutta, Anupam Dutta, Sanjeeb Kakati, Karuna K Barman, Dinesh Agarwal, Bikash Bhattacharjee, Neelakshi Bhattacharyya, Rahul Neog, Reema Nath, Chittaranjan S Yajnik

**Affiliations:** 1 Department of Medicine, Assam Medical College and Hospital, Dibrugarh, IND; 2 Diabetes and Endocrinology, Barman Diabetes Specialities, Guwahati, IND; 3 Department of Medicine, Marwari Hospitals, Guwahati, IND; 4 Diabetes and Endocrinology, Sun Valley Hospital, Guwahati, IND; 5 Multidisciplinary Research Unit, Assam Medical College and Hospital, Dibrugarh, IND; 6 Department of Microbiology, Assam Medical College and Hospital, Dibrugarh, IND; 7 Diabetes and Endocrinology, KEM Hospital, Pune, IND

**Keywords:** autoimmune diabetes, glutamic acid decarboxylase (gad) antibodies, latent autoimmune diabetes in adults (lada), northeast india, young-onset diabetes

## Abstract

Background: Latent autoimmune diabetes in adults (LADA) is a distinct form of diabetes characterized by autoimmune β-cell destruction with a slower progression compared to classical type 1 diabetes. It is frequently misclassified as type 2 diabetes, particularly in younger individuals, leading to suboptimal management. The presence of anti-glutamic acid decarboxylase (GAD) antibodies is a key diagnostic marker for LADA. However, data on the prevalence and distribution of GAD antibodies among young-onset diabetes patients in Northeast India are limited.

Methods: This retrospective cross-sectional study was conducted at the Multidisciplinary Research Unit of a tertiary care center in Northeast India using archived serum samples from the Indian Council of Medical Research (ICMR)-funded PHENOEINDY-2 (Phenotyping North East Indian Young Type 2 Diabetes) cohort study (2017-2021). A total of 149 individuals diagnosed with diabetes before 40 years of age were included based on the availability of adequate stored serum samples. Serum anti-GAD antibody levels were measured using a standardized enzyme-linked immunosorbent assay (ELISA). Participants were categorized as GAD-negative (<5 U/mL) or GAD-positive (≥5 U/mL), the latter considered suggestive of an autoimmune diabetes phenotype. Descriptive analyses were performed to determine the prevalence and distribution of GAD antibody levels.

Results: The overall mean GAD antibody level was 3.17 U/mL, with a median of 1.07 U/mL and a wide range (0.26-109.15 U/mL), demonstrating a markedly right-skewed distribution (skewness 7.52; kurtosis 63.29). Fourteen patients (9.4%) were classified as GAD-positive, indicating a probable diagnosis of LADA. The GAD-negative group (n=135) showed a tightly clustered distribution with low variability (mean 0.95 U/mL; SD 0.54 U/mL), consistent with non-autoimmune diabetes. In contrast, the GAD-positive group exhibited substantial heterogeneity (mean 24.58 U/mL; SD 29.67 U/mL), with several individuals demonstrating markedly elevated antibody levels exceeding 70-100 U/mL. Visual analyses confirmed a clear separation between the two groups, with compact clustering in the GAD-negative cohort and a wide dispersion in the GAD-positive subgroup.

Conclusion: Approximately 1 in 10 young-onset diabetes patients in this cohort demonstrated GAD positivity, suggesting that autoimmune diabetes represents an important subset of young-onset diabetes in Northeast India. These findings provide preliminary epidemiological evidence on the prevalence of GAD positivity in this population and support the need for larger prospective studies incorporating detailed clinical phenotyping, metabolic follow-up, and evaluation of the clinical utility of GAD antibody testing to improve diagnostic classification and patient management.

## Introduction

Latent autoimmune diabetes in adults (LADA) is a form of diabetes that straddles the characteristics of both type 1 and type 2 diabetes mellitus. Typically manifesting in adults over 30 years of age, LADA is characterized by the presence of autoantibodies against pancreatic islet cells, particularly glutamic acid decarboxylase antibodies (GADAs), and a gradual progression towards insulin dependence. Unlike the rapid onset of type 1 diabetes, LADA patients often do not require insulin therapy immediately, leading to frequent misdiagnosis as type 2 diabetes.

The prevalence of LADA varies across populations and is influenced by genetic and environmental factors. Studies have reported that among individuals diagnosed with type 2 diabetes, approximately 10% may have LADA, with this proportion increasing to 25% in those diagnosed before the age of 35 [1]. This significant subset underscores the importance of accurate diagnosis, as the management strategies for LADA differ markedly from those for type 2 diabetes.

Clinically, LADA patients present a unique phenotype. They are often younger at diagnosis and possess a lower body mass index (BMI) compared to typical type 2 diabetes patients. Additionally, LADA patients exhibit a more rapid decline in β-cell function, leading to earlier insulin dependence. The presence of GADAs serves as a crucial biomarker for distinguishing LADA from type 2 diabetes. In a study conducted in Finland, 9.3% of patients with type 2 diabetes were found to be GADA positive, and these patients demonstrated lower fasting C-peptide levels, indicating diminished β-cell function [2].

The pathophysiology of LADA involves an autoimmune response against pancreatic β-cells, similar to type 1 diabetes. However, the autoimmune attack in LADA is less aggressive, resulting in a slower progression to insulin dependence. Genetic predisposition plays a role, with certain HLA genotypes being more prevalent in LADA patients. Environmental factors, such as viral infections and lifestyle, may also contribute to disease onset.

Geographical variations in LADA prevalence have been observed. For instance, a study from Northern India reported a low prevalence of GAD antibodies among adults with type 2 diabetes, suggesting regional differences in autoimmune diabetes incidence [3]. Conversely, research from Western Finland identified a higher prevalence of GAD antibodies among Type 2 diabetes patients, highlighting the influence of genetic and environmental factors on different populations [2].

Early and accurate diagnosis of LADA is imperative for optimal patient management. Misclassification as type 2 diabetes can lead to inappropriate treatment strategies, such as the use of oral hypoglycemic agents that may not be effective. Identifying GAD antibodies in patients can aid in diagnosing LADA, allowing for the timely initiation of insulin therapy, which has been shown to preserve residual β-cell function and improve glycemic control [4].

## Materials and methods

This retrospective observational study was conducted at the Multidisciplinary Research Unit (MRU) of Assam Medical College and Hospital, a tertiary care center in Northeast India. The study aimed to evaluate the prevalence of anti-glutamic acid decarboxylase autoantibodies among young-onset diabetes patients using archived serum samples collected as part of the Indian Council of Medical Research (ICMR)-funded PHENOEINDY-2 (Phenotyping North East Indian Young Type 2 Diabetes) project between 2017 and 2021 [5].

A total of 149 individuals (90 male and 59 female patients) with diabetes diagnosed before the age of 40 years were included in the analysis. The median age for male patients was 35.5 (31.9-39.1, 25th and 75th percentiles) years, and that for female patients was 36.1 (31.4-40, 25th and 75th percentiles) years. These participants were selected from a larger cohort of 300 patients enrolled in the parent study, based on the availability of adequate, well-preserved serum samples in the MRU biobank. Samples that were insufficient in volume (less than 50 microliters) or degraded (haemolysed and containing red blood cells) were excluded. Only participants who had provided prior consent for future biochemical analyses were included.

Serum anti-GAD antibody levels were quantified using a commercially available enzyme-linked immunosorbent assay (ELISA) spectrophotometer (Multiskan GO; Thermo Scientific), performed in accordance with the manufacturer’s instructions. Briefly, stored serum samples were thawed under controlled conditions and diluted appropriately before analysis. The samples were then incubated in microplate wells pre-coated with GAD antigen. Following incubation, a secondary enzyme-conjugated antibody was added, and a chromogenic substrate reaction was used for detection. The assay was performed following the instructions of the Glutamic Acid Decarboxylase Autoantibody (GADAb) ELISA kit (RSR Ltd., Cardiff, UK). The RSR GADAb ELISA kit achieved 98% (n=100) specificity and 92% (n=50) sensitivity (see the Appendices). Optical density was measured at 450 nm using a calibrated ELISA plate reader, and antibody concentrations were calculated using a standard calibration curve generated from known controls.

For analytical purposes, participants were categorized into two groups based on GAD antibody levels: GAD-negative (<5 U/mL) and GAD-positive (≥5 U/mL), the latter considered suggestive of an autoimmune diabetes phenotype consistent with latent autoimmune diabetes in adults. Descriptive statistical analyses were performed to summarize GAD antibody levels, including measures of central tendency (mean and median) and dispersion (standard deviation and range). The distribution of GAD antibody levels was further characterized using measures of skewness and kurtosis. The prevalence of GAD positivity was calculated as the proportion of participants with antibody levels ≥5 U/mL. Data were graphically represented using histograms, box plots, and scatter plots to illustrate the distribution and variability of GAD antibody levels.

The primary outcome of the study was the prevalence of elevated GAD antibody levels among young diabetic patients. All procedures were conducted in accordance with institutional ethical standards, and approval was obtained from the Institutional Ethics Committee of Assam Medical College and Hospital. All data were anonymized before analysis to ensure participant confidentiality.

## Results

A total of 149 young-onset diabetes patients were included in the study, comprising 90 (60.4%) male and 59 (39.6%) female participants. The median age of the participants was comparable between male and female participants (35.5 (IQR 31.9-39.1) years vs. 36.1 (IQR 31.4-40.0) years, respectively). Anthropometric assessment demonstrated a median BMI of 24.0 (IQR 20.9-26.4) kg/m² among male participants and 23.8 (IQR 20.6-27.0) kg/m² among female participants, indicating that the majority of participants were in the normal to overweight range. Glycemic control was suboptimal in both groups, with median HbA1c levels of 9.3% (IQR 7.4-11.5) in male participants and 9.0% (IQR 7.4-11.6) in female participants. Median fasting C-peptide concentrations were similar between sexes (1.2 (IQR 0.5-2.2) ng/mL in male participants and 1.2 (0.8-2.2) ng/mL in female participants), suggesting comparable residual β-cell function at baseline. A family history of diabetes was reported in 40 participants (26.8%), including 14 males and 26 females, while the remaining 109 participants had no documented family history of diabetes. Overall, the baseline characteristics indicated that the study population consisted predominantly of young adults with poor glycemic control but relatively preserved endogenous insulin secretion, providing an appropriate cohort for evaluating the prevalence of GAD antibody positivity and identifying individuals with a potential autoimmune diabetes phenotype (Table 1). Participants were stratified into GAD-negative (<5 U/mL) and GAD-positive (≥5 U/mL) groups.

**Table 1:** Baseline demographic and biochemical characteristics of the study population (N=149) BMI, body mass index; HbA1c, glycated hemoglobin; IQR, interquartile range

Variable	Male (n=90)	Female (n=59)	Overall (N=149)
Age (years), median (IQR)	35.5 (31.9–39.1)	36.1 (31.4–40.0)	35.8
BMI (kg/m²), median (IQR)	24.0 (20.9–26.4)	23.8 (20.6–27.0)	23.9
HbA1c (%), median (IQR)	9.3 (7.4–11.5)	9.0 (7.4–11.6)	9.2
C-peptide (ng/mL), median (IQR)	1.2 (0.5–2.2)	1.2 (0.8–2.2)	1.2
Family history of diabetes, n (%)	40 (44.4%)	23 (39.0%)	63 (42.3%)
No family history of diabetes, n (%)	50 (55.6%)	36 (61.0%)	86 (57.7%)

Overall, the mean GAD antibody level in the cohort was 3.17 U/mL, with a median of 1.07 U/mL. The distribution demonstrated considerable variability (standard deviation: 11.20 U/mL), with values ranging from 0.26 to 109.15 U/mL. The data exhibited marked positive skewness (7.52) and high kurtosis (63.29), indicating a predominantly low-value distribution with a small subset of individuals showing markedly elevated antibody levels suggestive of an autoimmune phenotype.

Of the total cohort, 135 participants (90.6%) were classified as GAD-negative. In this group, the mean GAD antibody level was 0.95 U/mL, with a median of 1.03 U/mL. The variability was minimal (standard deviation: 0.54 U/mL), reflecting a tightly clustered distribution. The range of values remained well below the predefined threshold for GAD positivity, with a maximum value of 3.69 U/mL. The distribution in this subgroup demonstrated mild right skewness (skewness: 1.26), consistent with a relatively homogeneous population characteristic of non-autoimmune diabetes.

Among the study population, 14 individuals (9.4%) were classified as GAD-positive (≥5 U/mL), suggestive of an autoimmune diabetes phenotype consistent with latent autoimmune diabetes in adults. In this subgroup, the mean GAD antibody level was 24.58 U/mL, with a median of 12.88 U/mL. A high degree of variability was observed (standard deviation: 29.67 U/mL), reflecting a wide dispersion of antibody levels. The distribution was positively skewed (skewness: 2.31), indicating the presence of markedly elevated values in a subset of patients. The maximum recorded antibody level was 109.15 U/mL, further supporting significant autoimmune activity in this group. Compared to the GAD-negative group, the substantially greater variance observed in this subgroup highlights the heterogeneous nature of autoimmune diabetes within young-onset diabetic individuals.

The distribution of GAD antibody levels across the study population demonstrated a markedly right-skewed pattern, with the majority of participants exhibiting low antibody concentrations and a small subset showing disproportionately elevated values. This long right tail reflects the presence of individuals with significantly high GAD levels, consistent with an autoimmune diabetes phenotype.

**Figure 1:**
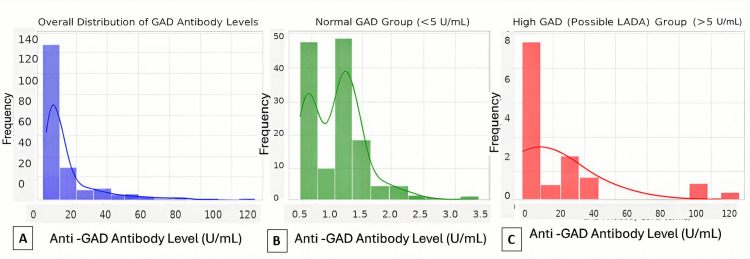
Histogram plots showing the distribution of anti-GAD antibody levels in the overall cohort (N=149), stratified by GAD status (A) The overall distribution is markedly left-skewed (mean 3.17 U/mL; median 1.07 U/mL; range 0.26-109.15 U/mL). (B) The GAD-negative group (<5 U/mL; n=135) demonstrates a narrow distribution (mean 0.95 U/mL), whereas (C) the GAD-positive group (≥5 U/mL; n=14) shows wide dispersion with markedly elevated values (mean 24.58 U/mL; maximum 109.15 U/mL), consistent with an autoimmune diabetes phenotype. GAD, glutamic acid decarboxylase; LADA, latent autoimmune diabetes in adults

Within the GAD-negative subgroup (<5 U/mL), antibody levels were tightly clustered, predominantly ranging between 0.5 and 2 U/mL. The distribution in this group was relatively compact, indicating low variability and a homogeneous profile typical of non-autoimmune diabetes. In contrast, the GAD-positive subgroup (≥5 U/mL) exhibited a wide dispersion of values, with several individuals demonstrating markedly elevated antibody levels, in some cases exceeding 70-100 U/mL. This substantial spread highlights the heterogeneity within the autoimmune subgroup and supports the presence of varying degrees of autoimmune activity among these patients (Figure 1).

Box plot analysis of GAD antibody levels demonstrated a clear distinction between the overall distribution and subgroup patterns. In the overall cohort, most values were concentrated below 5 U/mL, with a narrow interquartile range indicating that the majority of observations were tightly clustered in the lower range. However, several extreme outliers extending beyond 100 U/mL were observed, reflecting the presence of individuals with markedly elevated antibody levels suggestive of autoimmune diabetes.

**Figure 2:**
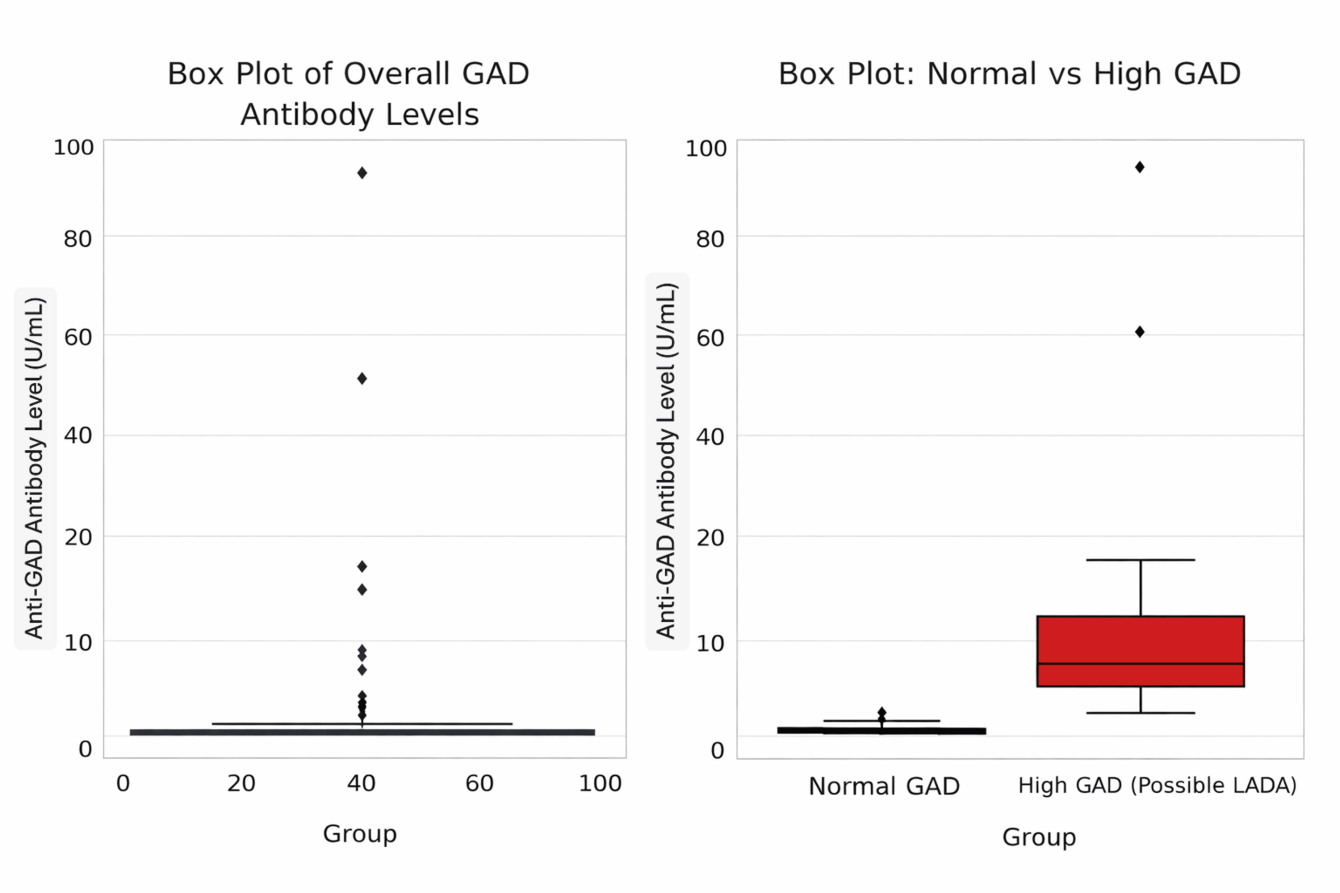
Box plots illustrating the distribution of anti-GAD antibody levels in the overall cohort (N=149), stratified by GAD status In the overall cohort, most values are concentrated below 5 U/mL with a narrow interquartile range, while several extreme outliers extend beyond 100 U/mL. The GAD-negative group (<5 U/mL; n=135) shows a compact distribution with minimal variability. In contrast, the GAD-positive group (≥5 U/mL; n=14) demonstrates a wide dispersion with a high variance and the presence of markedly elevated values (>70-100 U/mL), consistent with an autoimmune diabetes phenotype. GAD, glutamic acid decarboxylase; LADA, latent autoimmune diabetes in adults

When stratified by GAD status, the GAD-negative group (<5 U/mL) exhibited a compact distribution with minimal variability, with values consistently clustered within a narrow range. In contrast, the GAD-positive group (≥5 U/mL) showed a markedly wider dispersion, with substantial variability and the presence of extreme values exceeding 70-100 U/mL. This pronounced difference in spread highlights the heterogeneous nature of autoimmune diabetes, whereas the GAD-negative group demonstrates a relatively stable and homogeneous profile consistent with type 2 diabetes (Figure 2).

**Figure 3:**
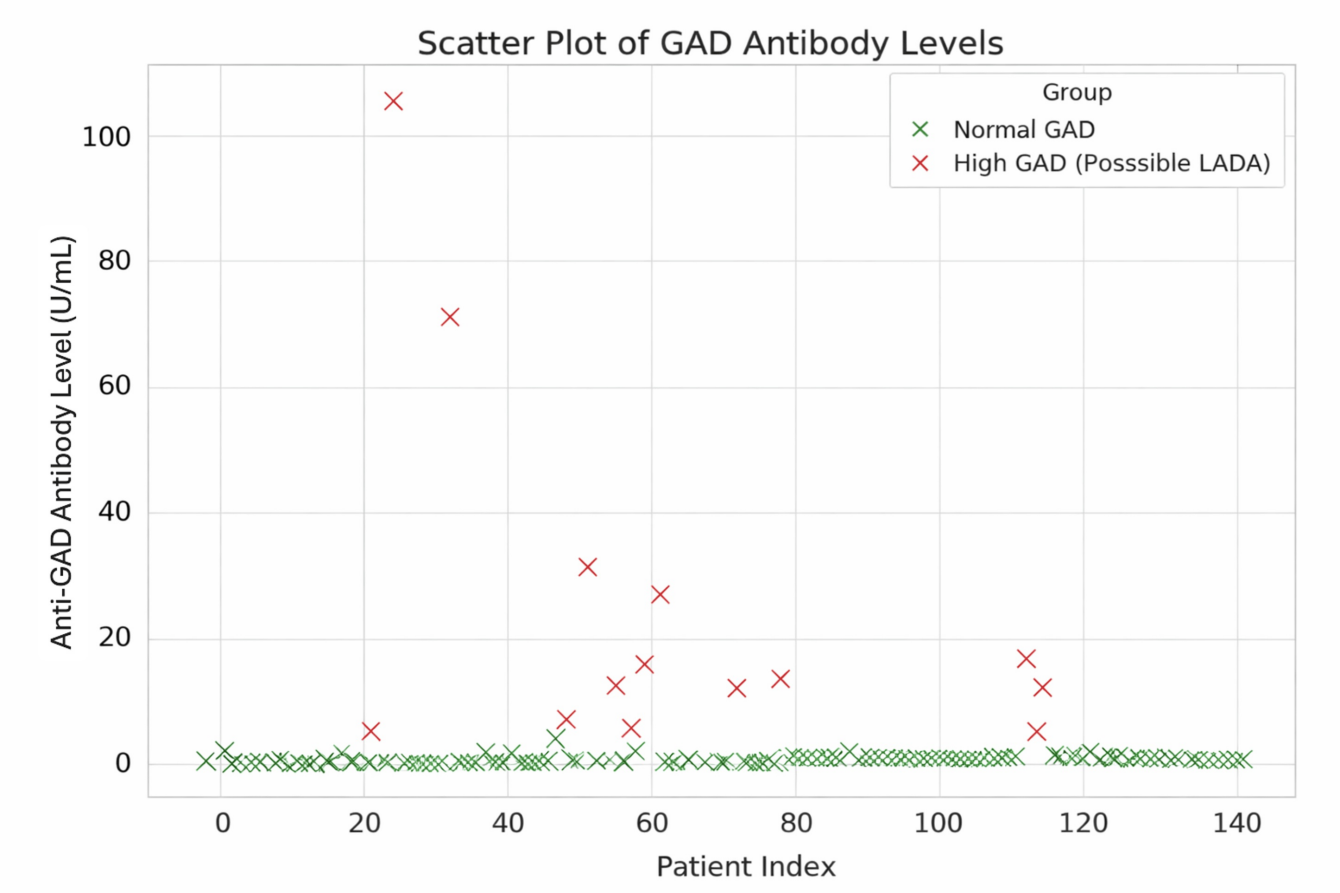
A scatter plot illustrating the distribution of anti-GAD antibody levels and phenotypic separation in young-onset diabetes (N=149) Each point represents an individual participant, with the GAD-negative group (<5 U/mL; n=135) shown in green and the GAD-positive group (≥5 U/mL; n=14) in red. The majority of patients are tightly clustered within the low antibody range (<5 U/mL), whereas GAD-positive individuals demonstrate a wider dispersion, including markedly elevated values (>70-100 U/mL). The distinct separation between the two groups highlights the presence of heterogeneous phenotypes, consistent with non-autoimmune and autoimmune forms of diabetes. GAD, glutamic acid decarboxylase; LADA, latent autoimmune diabetes in adults

Scatter plot analysis of anti-GAD antibody levels demonstrated distinct clustering patterns between groups. The majority of patients were clustered within the low antibody range (<5 U/mL), exhibiting a compact and homogeneous distribution. In contrast, the GAD-positive subgroup displayed a wide dispersion of values, with several individuals demonstrating markedly elevated levels exceeding 70-100 U/mL. This clear separation between the two groups highlights the presence of distinct phenotypic subsets within the cohort, supporting the identification of an autoimmune diabetes phenotype among GAD-positive individuals (Figure 3).

The GAD-negative group exhibited a tightly clustered distribution with low variability, consistent with conventional type 2 diabetes. In contrast, the GAD-positive group showed marked variability, with several individuals demonstrating markedly elevated antibody levels (>70-100 U/mL), suggestive of an autoimmune etiology. Histogram, box plot, and scatter plot analyses consistently demonstrated clear separation between the groups, highlighting a compact distribution in the GAD-negative cohort and a wider, heterogeneous spread among GAD-positive individuals.

## Discussion

This study aimed to determine the prevalence of LADA among young diabetic patients at Assam Medical College and Hospital. Our findings revealed that approximately 9.4% of the cohort exhibited elevated anti-GAD antibody levels (>5 U/mL), suggesting a significant subset of young diabetics may have LADA. The observed prevalence aligns with global data. A meta-analysis reported a worldwide LADA prevalence of 8.9% among diabetic individuals, with regional variations ranging from 2.3% to 18.9% [6,7]. Similarly, a study from China found a 9.2% prevalence of LADA among patients initially diagnosed with type 2 diabetes [8-10]. These consistent findings underscore the importance of considering LADA in the differential diagnosis of diabetes, especially in younger populations.

Our study also highlighted distinct clinical characteristics between LADA and type 2 diabetes patients. The LADA group demonstrated higher variability in GAD antibody levels, with some patients exhibiting markedly elevated titers (>70-100 U/mL). This variability is indicative of an autoimmune etiology, differentiating LADA from typical type 2 diabetes. Supporting this, research has shown that LADA patients often present with lower BMI and reduced β-cell function compared to their type 2 counterparts [11]. Additionally, the presence of GAD antibodies has been associated with a more rapid progression to insulin dependence [12,13].

The heterogeneity observed within the LADA population suggests varying degrees of autoimmune activity. Studies have proposed that higher GAD antibody titers correlate with more pronounced β-cell dysfunction and a quicker transition to insulin therapy [14,15]. This stratification emphasizes the need for personalized management approaches based on antibody levels and clinical presentation.

A notable strength of our study is that it provides one of the first estimates of GAD antibody positivity among young-onset diabetic patients from Northeast India, a region where data regarding LADA are scarce. By utilizing archived serum samples from the well-characterized ICMR-funded PHENOEINDY-2 cohort, we ensured standardized sample collection, storage, and laboratory analysis [5]. The use of a validated ELISA-based assay for GAD antibody estimation further enhanced the reliability of antibody measurements. These findings contribute valuable epidemiological data and highlight the presence of an autoimmune diabetes phenotype in a clinically relevant subset of young-onset diabetic patients.

However, several limitations should be acknowledged. The retrospective cross-sectional design limits causal inference and precludes assessment of the temporal progression of β-cell dysfunction or transition to insulin dependence. Selection bias may have occurred because only participants with adequate, archived serum samples were included. In addition, the study did not evaluate clinical outcomes, diagnostic accuracy of GAD antibody testing, insulin requirements, preservation of β-cell function, or the cost-effectiveness of GAD screening. Other autoimmune markers (such as IA-2 or ZnT8 antibodies), C-peptide levels, and genetic susceptibility markers were also not assessed. Therefore, while our findings demonstrate that approximately 1 in 10 young-onset diabetes patients exhibit GAD positivity, they should be interpreted as evidence of disease prevalence rather than as support for routine universal GAD antibody screening. Future prospective studies incorporating longitudinal clinical follow-up, metabolic assessment, and health-economic evaluation are needed to determine whether targeted GAD antibody testing in selected young-onset diabetes patients improves diagnostic classification and clinical outcomes.

## Conclusions

This study demonstrated that 9.4% of young-onset diabetes patients were positive for anti-glutamic acid decarboxylase antibodies, suggesting that latent autoimmune diabetes in adults represents a clinically relevant subset of young-onset diabetes in this population. The prevalence observed was comparable to that reported in international studies, and the marked variability in GAD antibody levels among GAD-positive participants further highlights the heterogeneous nature of autoimmune diabetes.

These findings provide epidemiological evidence supporting the consideration of GAD antibody testing in selected young-onset diabetes cases where autoimmune diabetes is clinically suspected, with the aim of improving diagnostic classification. However, this study did not evaluate the impact of GAD antibody testing on clinical outcomes, treatment decisions, or cost-effectiveness. Therefore, prospective studies incorporating detailed clinical, metabolic, immunological, and genetic characterization, together with long-term follow-up, are warranted to determine the clinical utility of targeted GAD antibody testing and to better define the epidemiology, natural history, and optimal management of LADA in the Indian population.

## Appendices

The assay was performed following the instructions of the Glutamic Acid Decarboxylase Autoantibody (GADAb) ELISA kit (RSR Ltd., Cardiff, UK). As per the manufacturer, the performance characteristics of the kit are as follows: in relation to clinical specificity and sensitivity, in the DASP 2005 study [16], the RSR GADAb ELISA kit achieved 98% (n=100) specificity and 92% (n=50) sensitivity.

**Table 2:** Inter-assay precision CV, coefficient of variation

Sample	GADAb value (u/mL) (n=20)	CV (%)
A	97	5.7
B	21	5.2
C	5.7	6.4

**Table 3:** Intra-assay precision CV, coefficient of variation

Sample	GADAb value (u/mL) (n=25)	CV (%)
1	97	7.3
2	20	8.5
3	7	3.5

## References

[1] Stenström G, Gottsäter A, Bakhtadze E, Berger B, Sundkvist G (2005). Latent autoimmune diabetes in adults: definition, prevalence, β-cell function, and treatment. Diabetes.

[2] Tuomi T, Carlsson A, Li H (1999). Clinical and genetic characteristics of type 2 diabetes with and without GAD antibodies. Diabetes.

[3] Sachan A, Zaidi G, Sahu RP, Agrawal S, Colman PG, Bhatia E (2015). Low prevalence of latent autoimmune diabetes in adults in northern India. Diabet Med.

[4] Buzzetti R, Di Pietro S, Giaccari A (2007). High titer of autoantibodies to GAD identifies a specific phenotype of adult-onset autoimmune diabetes. Diabetes Care.

[5] Dutta A, Dutta PK, Baruah SM (2025). Non-autoimmune diabetes in young people from Assam, India: the PHENOEINDY-2 study. Diabetologia.

[6] Magliano DJ, Boyko EJ, IDF Diabetes Atlas 10th Edition Scientific Committee (2026). IDF Diabetes Atlas [Internet], 10th Edition. https://www.ncbi.nlm.nih.gov/books/NBK581934/.

[7] Zhou Z, Xiang Y, Ji L (2013). Frequency, immunogenetics, and clinical characteristics of latent autoimmune diabetes in China (LADA China study): a nationwide, multicenter, clinic-based cross-sectional study. Diabetes.

[8] Hawa MI, Kolb H, Schloot N (2013). Adult-onset autoimmune diabetes in Europe is prevalent with a broad clinical phenotype: Action LADA 7. Diabetes Care.

[9] Liu L, Li X, Xiang Y (2015). Latent autoimmune diabetes in adults with low-titer GAD antibodies: similar disease progression with type 2 diabetes: a nationwide, multicenter prospective study (LADA China Study 3). Diabetes Care.

[10] Buzzetti R, Maddaloni E, Gaglia J, Leslie RD, Wong FS, Boehm BO (2022). Adult-onset autoimmune diabetes. Nat Rev Dis Primers.

[11] Pan N, Yang S, Niu X (2022). Latent autoimmune diabetes in adults and metabolic syndrome—a mini review. Front Endocrinol (Lausanne).

[12] Fourlanos S, Perry C, Stein MS, Stankovich J, Harrison LC, Colman PG (2006). A clinical screening tool identifies autoimmune diabetes in adults. Diabetes Care.

[13] Tuomi T, Santoro N, Caprio S, Cai M, Weng J, Groop L (2014). The many faces of diabetes: a disease with increasing heterogeneity. Lancet.

[14] Hwangbo Y, Kim JT, Kim EK (2012). Prevalence and clinical characteristics of recently diagnosed type 2 diabetes patients with positive anti-glutamic acid decarboxylase antibody. Diabetes Metab J.

[15] Zimmet PZ, Tuomi T, Mackay IR, Rowley MJ, Knowles W, Cohen M, Lang DA (1994). Latent autoimmune diabetes mellitus in adults (LADA): the role of antibodies to glutamic acid decarboxylase in diagnosis and prediction of insulin dependency. Diabet Med.

[16] Schlosser M, Mueller PW, Törn C (2010). Diabetes Antibody Standardization Program: evaluation of assays for insulin autoantibodies. Diabetologia.

